# Extensive Rectal Ischemia with Perforation: A Rare Case of Whole-Segment Ischemic Injury

**DOI:** 10.3390/reports9020106

**Published:** 2026-03-28

**Authors:** Jiun-Ru Juan, Jung-Cheng Kang, Ta-Wei Pu, Ruei-Yu Su, Yu-Chuan Chuang

**Affiliations:** 1 Department of Medical Education, Chung Shan Medical University Hospital, Taichung 402, Taiwan; smartynealson@yahoo.com.tw; 2 Department of Surgery, Division of Colon and Rectal Surgery, Taiwan Adventist Hospital, Taipei 105, Taiwan; jckang5534@gmail.com; 3 Division of Colon and Rectal Surgery, Department of Surgery, Songshan Branch, Tri-Service General Hospital, National Defense Medical Center, Taipei 105, Taiwan; tawei0131@gmail.com; 4 Division of Colon and Rectal Surgery, Department of Surgery, Tri-Service General Hospital, National Defense Medical Center, Taipei 114, Taiwan; 5 Department of Pathology, Tri-Service General Hospital, National Defense Medical Center, Taipei 114, Taiwan; rueiyusu@gmail.com; 6 Department of Pathology and Laboratory Medicine, Taoyuan Armed Forces General Hospital, Taoyuan 325, Taiwan; 7 Division of Gastroenterology, Department of Internal Medicine, Songshan Branch, Tri-Service General Hospital, National Defense Medical Center, Taipei 105, Taiwan

**Keywords:** septic shock, rectal ischemia, Hartmann’s procedure, hypertensive cardiovascular disease

## Abstract

**Background and Clinical Significance:** Extensive rectal ischemia is exceptionally rare due to the rectum’s robust vascular network, with segmental ischemia being more common. **Case Presentation:** We report the case of a 69-year-old female who presented with whole-segment rectal ischemia, encompassing the upper, mid, and lower rectum. This severe local ischemic event culminated in full-thickness perforation and extensive fecal peritonitis, which subsequently precipitated postoperative septic shock. The patient underwent emergency low anterior resection with Hartmann’s procedure and received intensive multidisciplinary postoperative care. **Conclusions**: In this case, we aimed to highlight the importance of early recognition, decisive surgical intervention, and the pathophysiological and diagnostic challenges in managing rare cases of whole-segment rectal ischemia.

## 1. Introduction and Clinical Significance

Rectal ischemia is a rare but serious condition, distinct from ischemic colitis, which commonly affects the left colon. The rectum’s extensive collateral blood supply usually protects it from ischemic injury [1,2,3]. However, systemic factors such as severe hypotension, thromboembolism, hypercoagulable states, and cardiovascular comorbidities can overwhelm this vascular network, leading to ischemic changes and, in severe cases, perforation [4,5,6,7]. In this case report, we discuss the diagnostic and management complexities of rectal ischemia in an older patient with comorbidities, including hypertension and cardiovascular disease. Unlike typical cases where systemic shock precedes bowel necrosis, this patient developed severe, spontaneous whole-segment rectal ischemia that led to full-thickness perforation and fecal peritonitis. This massive intra-abdominal infection then precipitated severe postoperative septic shock. While segmental ischemia, particularly of the upper rectum, is more commonly observed in systemic hypoperfusion scenarios, this case describes a rare instance of whole-segment rectal ischemia, challenging the conventional understanding of the rectum’s vascular resilience.

## 2. Case Presentation

### 2.1. Patient History

The patient, a 69-year-old female, had a medical history significant for hypertension managed with antihypertensive medication. Although the specific pre-admission antihypertensive agents were not distinctly itemized in the initial emergency records beyond confirming that she was under regular medication control, her hypertension was a chronic, long-standing condition. On preoperative day 1, she presented to the emergency department with severe lower abdominal pain, difficulty with defecation, and episodes of bloody stool. She reported occasional black, tarry stools but denied fever, chills, nausea, vomiting, or weight loss. She also had a history of controlled cardiovascular disease but no recent surgeries or thromboembolic events.

### 2.2. Initial Clinical Examination

On arrival, the patient was alert (Glasgow Coma Scale: E4M6V5) but hypertensive, with systolic blood pressure readings between 160 and 190 mmHg. Physical examination revealed mild tenderness in the lower abdomen. A digital rectal examination demonstrated blood on the glove without signs of hemorrhoids or any palpable mucosal defects or perforations. The rest of the physical examination, including neurological and cardiopulmonary assessments, was unremarkable.

### 2.3. Laboratory and Imaging Findings

Initial laboratory investigations showed significant leukocytosis (white blood cell count: 16.97 × 10^3^/μL), indicating an acute inflammatory response. Hemoglobin levels were initially within the normal range (14.1 g/dL) but subsequently declined rapidly to 7.7 g/dL on POD 3, suggesting active gastrointestinal bleeding. Platelet count decreased to 115 × 10^3^/μL, and C-reactive protein spiked to 42.77 mg/dL by POD 3, consistent with systemic inflammation. The patient’s N-terminal pro-B-type natriuretic peptide was mildly elevated at 106.6 pg/mL, suggesting cardiac strain likely due to underlying hypertension and sepsis.

**Figure 1:**
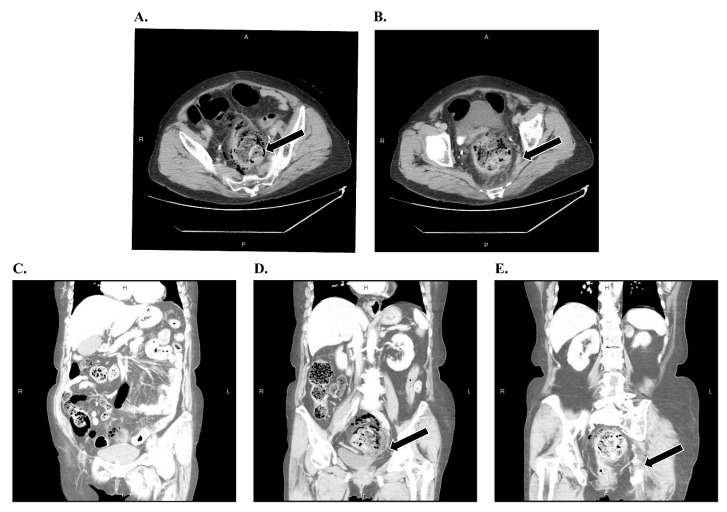
(**A**–**E**) Contrast-enhanced CT imaging demonstrating extraluminal gas (arrows) in the perirectal space, indicating full-thickness ischemic rectal perforation.

An abdominal and pelvic CT scan was performed in the emergency department prior to hospital admission on preoperative day 1. The images revealed free air surrounding the rectum (pneumoperitoneum), rectal wall thickening, and signs of ischemic changes (Figure 1). No evidence of mesenteric vessel thrombosis was observed. Given the severity of these findings, an emergency colorectal surgical consultation was requested.

### 2.4. Surgical Intervention

**Figure 2:**
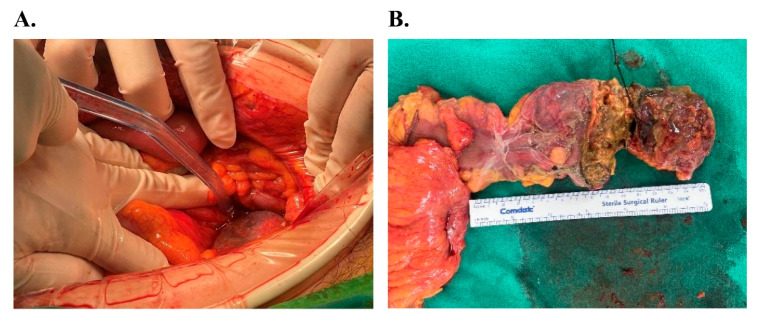
Intraoperative and macroscopic findings: (**A**) Low anterior resection (LAR) with Hartmann’s procedure. (**B**) Resected rectal segments displaying dark, edematous mucosa and transmural ischemic necrosis.

On POD 0, the patient underwent an exploratory laparotomy. Intraoperative findings revealed severe ischemic necrosis of the rectum with full-thickness perforation and extensive fecal peritonitis. The surgical team performed a low anterior resection (LAR) with Hartmann’s procedure, excising three segments of the rectum measuring 9.0 cm, 9.5 cm, and 3.0 cm. Gross examination of the resected specimen showed dark, edematous mucosa with ischemic changes extending to the serosal surface (Figure 2).

### 2.5. Pathological Findings

**Figure 3:**
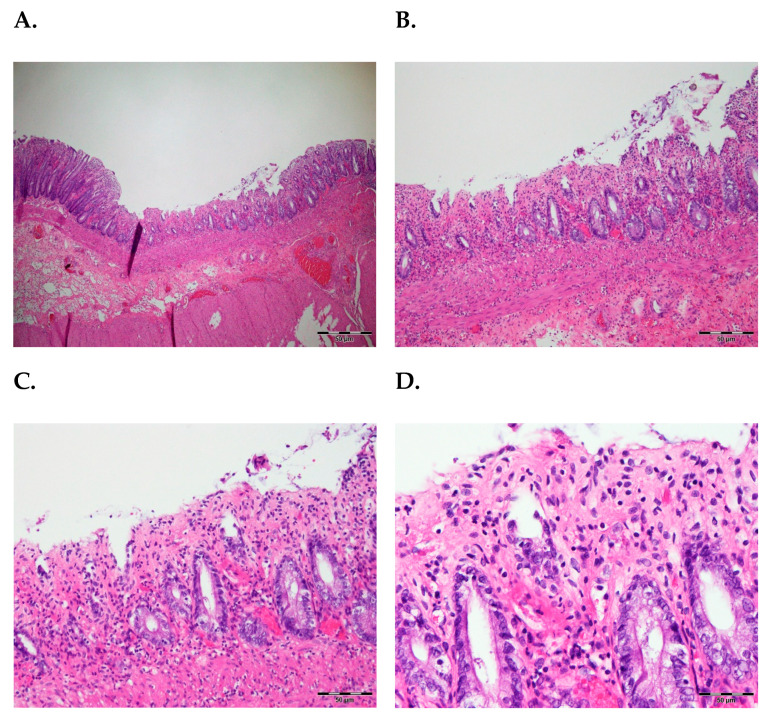
Histopathology of non-occlusive ischemia: (**A**,**B**) Rectal mucosa exhibiting sloughing, congestion, edema, and acute inflammation. (**C**,**D**) Intact arterial walls without thrombus.

Histopathological analysis confirmed severe rectal ischemia characterized by mucosal sloughing, congestion, and extensive edema. Acute inflammation was evident, with infiltration of neutrophils extending to the serosal layer, consistent with peritonitis. No thrombus was identified in the mesenteric arteries, suggesting a non-occlusive ischemic process likely secondary to chronic microvascular compromise from her underlying hypertensive cardiovascular disease (Figure 3).

### 2.6. Postoperative Course

The patient was admitted to the intensive care unit (ICU) due to septic shock following surgery. She received aggressive intravenous fluid resuscitation and albumin supplementation. Because she remained hypertensive (systolic blood pressure of 160–190 mmHg) despite her critical condition, a Nicardipine pump was utilized to actively and strictly control her blood pressure while maintaining adequate perfusion. Extubation was attempted on POD 1, followed by respiratory support with a Venturi mask, which was gradually weaned to a nasal cannula by POD 6.

Intraoperative hematuria was noted, prompting urological consultation. The hematuria was suspected to be related to sepsis-induced coagulopathy. A three-way Foley catheter with saline irrigation was placed, and the hematuria resolved by POD 3. Dietary progression was cautious, starting with nothing by mouth status and nasogastric decompression, transitioning to a clear liquid diet on POD 3, and advancing to a full liquid diet by POD 7. During the postoperative period, the patient developed a skin rash with bullae formation over her right armpit; a dermatology consultation diagnosed a herpes zoster infection, which was treated with topical medications and Medovir.

On POD 14, the Jackson–Pratt (J-P) drain output became brownish, suggesting a possible intra-abdominal infection. While the ascitic fluid culture obtained on POD 15 showed no bacterial growth, an earlier anaerobic wound culture obtained on POD 8 grew Peptoniphilus asaccharolyticus and unidentified anaerobic Gram-negative bacilli, confirming the presence of infection. The laparotomy wound exhibited signs of dehiscence with sloughy discharge and a foul odor, requiring debridement and a rectus local flap closure on POD 15. Following this procedure, the patient maintained acceptable hemodynamics, good wound healing, and well-tolerated oral intake. She was subsequently discharged in a stable condition with planned outpatient department (OPD) follow-up. Because this is a recent case, long-term follow-up regarding stoma reversal, functional recovery, and overall quality of life is not yet available.

## 3. Discussion

### 3.1. Unique Insights from This Case

In this case, we present a rare occurrence of whole-segment rectal ischemia, involving the upper, mid, and lower rectum, which is typically protected by an extensive vascular supply. Rectal ischemia is usually segmental; therefore, the rarity of lower rectal ischemia makes this case particularly notable [8,9]. The widespread ischemia observed in our patient initially developed prior to any hemodynamic instability, likely driven by her underlying hypertensive cardiovascular disease rather than an initial state of shock. This extensive ischemic injury overwhelmed the vascular defenses of the lower rectum, leading to full-thickness perforation and severe intra-abdominal fecal peritonitis, which subsequently precipitated the postoperative septic shock.

Pathophysiologically, rectal ischemia can be occlusive or non-occlusive. In this case, the absence of a thrombus points to non-occlusive mesenteric ischemia (NOMI) [5,6,10]. While NOMI is most frequently caused by systemic hypoperfusion or shock in critically ill patients, our patient presented to the emergency department alert and severely hypertensive. This chronic microvascular compromise likely overwhelmed the vascular defenses of the lower rectum, leading to spontaneous whole-segment ischemia and subsequent full-thickness perforation. The profound systemic hypotension and septic shock observed in this patient developed only after the extensive intra-abdominal fecal peritonitis occurred, acting as a secondary catastrophic complication rather than the primary cause of the ischemia [11].

### 3.2. Diagnostic Challenges and Advances

The diagnosis of rectal ischemia is challenging, as its clinical presentation often mimics other gastrointestinal disorders, such as diverticulitis or ulcerative colitis [12]. The diagnosis of rectal ischemia relies on a triad of radiologic, intraoperative, and histologic criteria. Radiologically, contrast-enhanced CT demonstrates rectal wall thickening, pneumoperitoneum, and decreased mucosal enhancement [13,14]. In this case, embolic disease was definitively ruled out because histopathological analysis of the resected specimen confirmed the absence of any thrombus in the mesenteric arteries. Radiation proctitis was excluded, as the patient had no prior history of pelvic radiation therapy. Additionally, stercoral perforation was considered but dismissed, as there was no evidence of severe fecal impaction or stercoral ulcers noted during the initial digital rectal examination or during the intraoperative exploratory laparotomy. Finally, vasculitis was deemed highly unlikely given the patient’s lack of prior autoimmune history and the complete absence of systemic rheumatologic symptoms, such as arthralgia or joint swelling, throughout her clinical course. CT findings were crucial in identifying the extent of ischemic injury and guiding the decision for emergency surgery [13,14]. Intraoperatively, findings include full-thickness ischemic necrosis and edematous, dark mucosa extending to the serosa. Histologically, it is confirmed by mucosal sloughing, acute inflammatory infiltration, and the absence of arterial thrombi, which distinguishes it from occlusive disease [15].

Recent advances, including endoscopic Doppler ultrasound and perfusion CT, have shown promise in assessing mesenteric blood flow in real-time. These techniques may facilitate earlier diagnosis and improve outcomes by enabling timely intervention. Despite their potential, these technologies are not yet widely available and require further clinical validation [16].

### 3.3. Multidisciplinary Management Approach

Effective management of severe rectal ischemia requires a multidisciplinary approach, particularly in cases complicated by perforation and peritonitis [17]. In our patient, the decision to perform an LAR with Hartmann’s procedure was driven by the need to excise necrotic tissue and control peritoneal contamination. This surgical approach is supported by recent systematic reviews, which have shown improved survival rates when early resection is performed before the onset of multi-organ failure [18].

Postoperative care is complex, involving close collaboration among critical care, surgical, urological, and dermatological teams. In our case, complications such as septic shock, coagulopathy, and wound dehiscence required a coordinated effort [19]. The occurrence of herpes zoster in the postoperative period may indicate an immunocompromised state, highlighting the need for vigilant monitoring and prompt treatment of secondary infections in critically ill patients [20].

### 3.4. Emerging Therapeutic Strategies

Emerging therapeutic strategies for rectal ischemia focus on optimizing systemic perfusion and addressing coagulopathic states. Aggressive fluid resuscitation, vasopressor support, and albumin supplementation are critical in restoring hemodynamic stability and enhancing mesenteric blood flow [16,21]. In our patient, Nicardipine effectively controlled blood pressure while maintaining adequate perfusion, aligning with guidelines recommending hemodynamic optimization to prevent further ischemic damage [22].

### 3.5. Prognostic Considerations

The prognosis of rectal ischemia is significantly influenced by the speed of diagnosis and intervention. Delayed recognition can lead to irreversible tissue damage, perforation, and multi-organ failure. Early surgical resection, as in this case, improves survival outcomes by removing necrotic tissue and preventing further sepsis [23,24]. However, postoperative complication risks remain high, particularly in patients with extensive ischemic injury and underlying cardiovascular diseases [24,25]. This case highlights the importance of early recognition, timely surgical intervention, and a coordinated, multidisciplinary approach to managing severe rectal ischemia, particularly in older patients with cardiovascular risk factors. However, as a single retrospective case report, this study is limited by its inherent lack of generalizability, absence of long-term follow-up, and lack of advanced vascular imaging.

## 4. Conclusions

In summary, this case highlights the rare presentation of whole-segment rectal ischemia. The involvement of all rectal segments underscores the profound impact that chronic microvascular compromise and hypertensive cardiovascular disease can have in overwhelming the rectum’s robust vascular resilience. Early diagnosis via contrast-enhanced CT and prompt surgical intervention with a low anterior resection and Hartmann’s procedure were critical in managing this life-threatening perforation. Following a complex postoperative course complicated by septic shock, sepsis-induced coagulopathy, wound dehiscence, and secondary infections, the patient was successfully stabilized and discharged, highlighting the critical importance of a coordinated, multidisciplinary approach in optimizing outcomes. Further research is needed to better understand the pathophysiological thresholds that disrupt the rectum’s vascular defenses and to explore innovative strategies for managing severe rectal ischemia.

## Data Availability

The data presented in this study are available upon request from the corresponding author. The data are not publicly available due to privacy or ethical restrictions.

## Abbreviations

The following abbreviations are used in this manuscript:

CT	computed tomography
LAR	low anterior resection
ICU	intensive care unit
POD	postoperative day
J-P	Jackson–Pratt
